# Nomogram for predicting olfactory disorder in obstructive sleep apnea: A retrospective study based on a multicenter database

**DOI:** 10.1371/journal.pone.0318145

**Published:** 2025-03-27

**Authors:** Jiajia Dong, Xiao Yu, Yazhu Liang, Honglei Zhang, Haili Sun, Rui Guo

**Affiliations:** 1 Department of Otorhinolaryngology Head and Neck Surgery, Beijing Tiantan Hospital, Capital Medical University, Beijing, China; 2 Department of Otorhinolaryngology Head and Neck Surgery, Air Force Medical Center, Air Force Medical University, Beijing, China; 3 Graduate School of China Medical University, Shenyang, China; 4 Department of Otolaryngology, Beijing Anzhen Hospital, Capital Medical University, Beijing, China; 5 Key Laboratory of Upper Airway Dysfunction-related Cardiovascular Diseases, Beijing Institute of Heart, Lung and Blood Vessel Diseases, Beijing, China; Sapienza University of Rome: Universita degli Studi di Roma La Sapienza, ITALY

## Abstract

**Objective:**

Obstructive sleep apnea (OSA) increases the risk of olfactory disorder (OD), which may serve as an early warning of adverse health consequences. In this study, we aimed to develop and validate a nomogram for early detection of OD in patients with OSA.

**Methods:**

We retrospectively analyzed 125 patients with OSA at Beijing Anzhen Hospital for the development and internal validation of the nomogram. For external validation, 30 patients with OSA were recruited from the Air Force Medical Center. The included participants completed polysomnography (PSG) and the Sniffin’ Sticks test. Patients with OSA were divided into two groups: OSA with OD and OSA without OD.

**Results:**

The nomogram included age, sex, and time spent with oxygen saturation below 90%. The area under the receiver operating characteristic curve of the nomogram was 0.814 (95% confidence interval [CI]: 0.673–0.955) for the internal validation group, and 0.778 (95% CI: 0.601–0.955) for the external validation group. The nomogram exhibited excellent discrimination and calibration, showing substantial benefits in clinical applications.

**Conclusion:**

The present nomogram developed based on clinical characteristics and PSG features can serve as a convenient tool for clinicians to detect OD in OSA, aiding in patient stratification and personalized treatment.

## Introduction

Obstructive sleep apnea (OSA) is characterized by repetitive apnea and hypopnea during sleep, associated with intermittent hypoxia and arousal from sleep [1]. There is growing interest in the relationship between OSA and several adverse health consequences, including diabetes, cognitive decline, cardiovascular diseases, and olfactory disorder (OD) [2–6]. OD is prevalent among people with OSA [6–10]. OD not only results in reduced quality of life, nutritional imbalances, and diminished social functioning but is also associated with adverse outcomes such as frailty and mortality [11–14]. Our previous work suggested a link between olfactory disorder (OD) and cognitive decline in patients with OSA [15]. OD is frequently associated with alterations in the hippocampus and entorhinal cortex, areas that are also implicated in the development of dementia [16–18]. Assessment of olfaction can predict dementia conversion in individuals with mild cognitive impairment as well as future cognitive decline among adults initially classified as cognitively normal [19,20]. Therefore, it is important to incorporate olfactory assessment into routine evaluation for OSA to enhance the assessment process.

The most commonly used olfactory assessment tool in the clinical setting is the University of Pennsylvania Scent Identification Test, which comprises 40 odor identification questions, and the Sniffin’ Sticks test for evaluating odor identification, discrimination, and detection thresholds [21]. These two psychophysical assessments involve prolonged administration and supervision by a trained proctor, making them labor-intensive and time-consuming. Hence, an urgent need remains for a rapid screening tool for olfactory assessment.

To address this issue, we aimed to develop and validate a nomogram to predict OD in patients with OSA. We used comprehensive clinical sleep laboratory data, including the results of polysomnography (PSG) and data on associated comorbidities. These data elements were specifically chosen for their potential to provide insight into the underlying mechanisms of OD in patients with OSA. Our approach integrates these factors into a nomogram, aiming to improve predictive accuracy while remaining applicable in routine clinical use.

## Materials and methods

### Participants and study design

**Fig 1:**
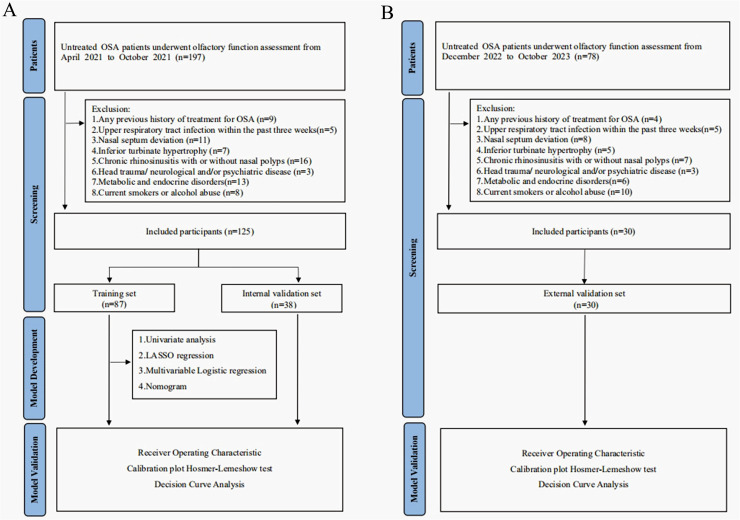
Flowchart diagram of research strategy in the Beijing Anzhen Hospital (A) and Air Force Medical Center (B). OSA, obstructive sleep apnea.

We conducted a retrospective analysis among patients with OSA who underwent olfactory evaluation at Beijing Anzhen Hospital between April 2021 and October 2021. The included participants were randomly allocated into a training set and an internal validation set at a 7:3 ratio. The flowchart diagram of the research strategy is presented in Fig 1A. Patients with OSA who attended the Air Force Medical Center from December 2022 to October 2023 were designated as the external validation set (Fig 1B). The study population comprised patients with newly diagnosed OSA, based on the results of overnight PSG. The exclusion criteria were as follows: (1) any previous history of treatment for OSA, including surgical intervention or CPAP use; (2) upper respiratory tract infection within the past 3 weeks, including patients with a history of post-viral olfactory dysfunction; (3) nasal septum deviation; (4) inferior turbinate hypertrophy; (5) chronic rhinosinusitis with or without nasal polyps; (6) head trauma and neurological and/or psychiatric disease; (7) metabolic and endocrine disorders; (8) current smokers (more than 10 cigarettes per day) or alcohol abuse (more than 210 g ethanol per week).

We used a training set comprising 70% of patients with OSA from Beijing Anzhen Hospital, which was used to construct a predictive model using a nomogram to distinguish patients with OSA with and without OD. The internal validation set comprised the remaining 30% of patients with OSA from Beijing Anzhen Hospital, which was used to assess and validate the diagnostic performance of the developed model. In addition, an independent external validation set comprising 30 patients with OSA from the Air Force Medical Center was incorporated to further validate the predictive model.

The data used in this study were accessed and analyzed between November 2023 and January 2024. The study followed the ethical principles of the Helsinki Declaration. This study was approved by the Ethics Committee of the Beijing Anzhen Hospital (No. 2022063X) and Air Force Medical Center (No. 2023-23-S01). All patients were informed in advance about the purpose of this study and signed informed consent forms.

### Data collection

We collected demographic and clinical characteristics of all patients, including age; sex; body mass index; neck circumference; and history of hypertension, hyperlipidemia, or coronary heart disease All patients underwent comprehensive otolaryngological examination and completed olfactory function assessment and sleep questionnaires, followed by overnight PSG. The sleep questionnaires included the Epworth Sleepiness Scale and Subjective Perception of Sleep Score.

### Polysomnography (PSG)

Participants underwent sleep monitoring for more than 7 consecutive hours in a sleep laboratory using standard PSG equipment (Siesta, Compumedics, Melbourne, Australia). OSA was diagnosed as an apnea/hypopnea index (AHI) ≥  5 events per hour. AHI was defined as the average number of apnea and hypopnea events per hour of sleep. Manual scoring was performed according to Version 2.4 published by the American Academy of Sleep Medicine [22]. We collected the following data: AHI, central apnea index, obstructive apnea index, mixed apnea index, hypopnea index, time spent with oxygen saturation below 90% (T90%), oxygen desaturation index (ODI), average duration of hypopnea, average duration of obstructive apnea, lowest oxygen saturation (SpO2%), and average blood oxygen.

### Olfactory function assessment

All participants were requested not to smoke, eat, or drink anything other than water for 15 min prior to the test. Participants’ binaural olfactory functions were assessed using a validated Sniffin’ Sticks test (Burghart Instruments, Germany) in a quiet and well-ventilated room. Following the manufacturer’s instructions, three separate tests were conducted: odor thresholds, odor discrimination, and odor identification. The results of all three tests were combined to calculate the threshold–discrimination–identification (TDI) score. All tests were performed by the same investigator. As per the odor test battery [23], normosmia was defined as a TDI score of 30.75 or above, and hyposmia was defined as a TDI score below 30.75, and anosmia was defined as TDI score below 16.

### Statistical analysis

The normality of continuous variables was assessed using the Kolmogorov–Smirnov test. The variables are described as mean ±  standard deviation or median with interquartile range (25th–75th), and were analyzed using either a t-test or Mann–Whitney U test. Binary categorical variables are expressed as number and percentage (n, %), and comparisons were conducted using either the chi-square test or Fisher’s exact test. In the training set, univariate analysis was used to screen for factors of difference between patients with OSA with and without OD. Statistical significance was defined as *P* <  0.05. We used IBM SPSS software version 25.0 in the analysis.

Least absolute shrinkage and selection operator (LASSO) regression analysis was performed in R software (version 4.2.0) using the glmnet package. LASSO regression was used to identify the optimal combination of factors affecting olfactory function in the training set using 10-fold cross-validation and lambda 1se. The variables selected in LASSO regression analysis were applied in multivariable logistic regression to further determine the factors influencing olfactory disorder in patients with OSA. Multivariable logistic regression was analyzed using the glm package. Statistical significance was defined as *P* <  0.05.

Influencing factors in multivariable logistic regression were adopted to establish a prediction model through a nomogram in the rms package. The receiver operating characteristic (ROC) curve and area under the ROC curve (AUC) were quantified using the pROC package to determine the discrimination capacity of the model group and validation groups. The calibration curve plot and Hosmer–Lemeshow test were used to evaluate the consistency between the nomogram-predicted and the actual probabilities, using the rms package and Resource Selection package, respectively. Decision curve analysis (DCA) was performed using the rmda package to evaluate clinical efficacy.

## Results

### Baseline characteristics

A total of 125 patients with OSA from Beijing Anzhen Hospital were included in this study and were randomly divided into the training set (n =  87) and internal validation set (n =  38). In addition, 30 patients from the Air Force Medical Center were included in the external validation set. The demographic and clinical characteristics of study participants in the three groups are shown in Table 1.

**Table 1:** The baseline characteristics of the enrolled patients in the training set and validation sets.

Characteristics	Study population (n = 155)	Training set (n = 87)	Internal validation set (n = 38)	External validation set (n = 30)	*P*
**Age**	53.0(48.5, 57.0)	53.0(48.0, 57.5)	54.0(41.2, 57.8)	53.0(52.0, 53.0)	0.743
**Gender**					0.241
** Female**	37 (23.9%)	21 (24.1%)	6 (15.8%)	10 (33.3%)	
** Male**	118 (76.1%)	66 (75.9%)	32 (84.2%)	20 (66.7%)	
**BMI**	27.0(25.1, 29.6)	26.8(25.0, 29.4)	27.0(24.9, 30.5)	27.6(25.8, 29.9)	0.720
**NC**	40.0(38.0, 43.0)	40.0(38.0, 42.0)	40.5(38.0, 43.8)	41.0(38.0, 43.8)	0.370
**SPSS**	7.0(5.00, 8.00)	7.0(5.00, 8.00)	7.0(5.00, 8.00)	7.0(5.00, 7.00)	0.565
**ESS**	11.3 ± 5.35	11.3 ± 5.17	11.9 ± 5.19	10.3 ± 6.06	0.452
**CAI**	0.2(0.10, 0.60)	0.2(0.10, 0.70)	0.1(0.10, 0.50)	0.2(0.20, 0.67)	0.126
**OAI**	7.8(2.15, 18.8)	7.2(2.45, 18.8)	7.1(1.25, 17.1)	11.6(2.72, 21.9)	0.547
**MAI**	0.2(0.10, 1.00)	0.10(0.10, 0.80)	0.10(0.10, 0.73)	0.60(0.00, 1.48)	0.776
**HI**	10.9(6.80, 18.4)	10.6(6.40, 17.0)	11.1(8.57, 21.4)	14.4(6.82, 19.0)	0.492
**AHI**	24.7(15.6, 44.6)	26.0(15.6, 43.4)	24.5(15.5, 46.6)	24.7(16.4, 44.7)	0.932
**T90%**	4.3(0.50, 10.2)	4.8(0.30, 9.85)	4.5(0.52, 14.4)	3.8(1.25, 6.20)	0.827
**ODI**	24.6(13.2, 43.9)	25.8(13.8, 45.0)	24.8(11.6, 43.8)	22.8(13.2, 35.8)	0.917
**Minimum SpO2**	80.0(75.0, 86.0)	80.0(75.5, 86.0)	79.0(74.2, 84.8)	81.0(76.5, 86.0)	0.391
**Average blood oxygen**	94.0(93.0, 95.0)	94.0(93.0, 95.0)	94.0(93.0, 95.0)	95.0(93.6, 95.7)	0.077
**ADH**	29.1(25.8, 33.2)	29.7(26.2, 33.4)	29.2(25.4, 34.3)	28.0(22.9, 30.8)	0.227
**ADOA**	25.2(20.2, 31.3)	25.0(20.5, 32.2)	28.4(20.4, 34.4)	25.0(19.2, 26.8)	0.084
**Hypertension**					0.497
** Yes**	69 (44.5%)	37 (42.5%)	20 (52.6%)	12 (40.0%)	
** No**	86 (55.5%)	50 (57.5%)	18 (47.4%)	18 (60.0%)	
**Coronary heart disease**					0.900
** Yes**	27 (17.4%)	15 (17.2%)	6 (15.8%)	6 (20.0%)	
** No**	128 (82.6%)	72 (82.8%)	32 (84.2%)	24 (80.0%)	
**Hyperlipidemia**					0.213
** Yes**	45 (29.0%)	27 (31.0%)	7 (18.4%)	11 (36.7%)	
** No**	110 (71.0%)	60 (69.0%)	31 (81.6%)	19 (63.3%)	
**TDI**	28.5(25.5, 31.4)	28.2(24.5, 31.5)	28.5(25.1, 30.9)	28.5(27.2, 31.4)	0.393
** T**	5.5(4.25, 7.50)	5.5(3.75, 7.75)	5.0(2.94, 6.88)	6.0(5.50, 6.50)	0.092
** D**	11.0(9.00, 12.0)	11.0(9.00, 12.0)	11.0(10.0, 12.0)	11.0(9.00, 12.0)	0.301
** I**	12.0(11.0, 13.0)	12.0(11.0, 13.0)	12.0(11.0, 13.0)	12.0(11.0, 13.0)	0.258

Abbreviation: BMI: body mass index; NC: neck circumference; SPSS: Subjective perception of sleep score; ESS: Epworth sleep scale; CAI: central apnea index; OAI: obstructive apnea index; MAI: mixed apnea index; HI: hypopnea index; AHI: apnea hypopnea index; T90%: time spent with oxygen saturation below 90%; ODI: oxygen desaturation index; ADH: average duration of hypopnea; ADOA: average duration of obstructive apnea; TDI: threshold discrimination identification; T:threshold; D:discrimination; I: identification. *P <* 0.05 is statistically significant.

### Univariate analysis and LASSO regression in patients who have OSA with OD and non-OD

**Fig 2:**
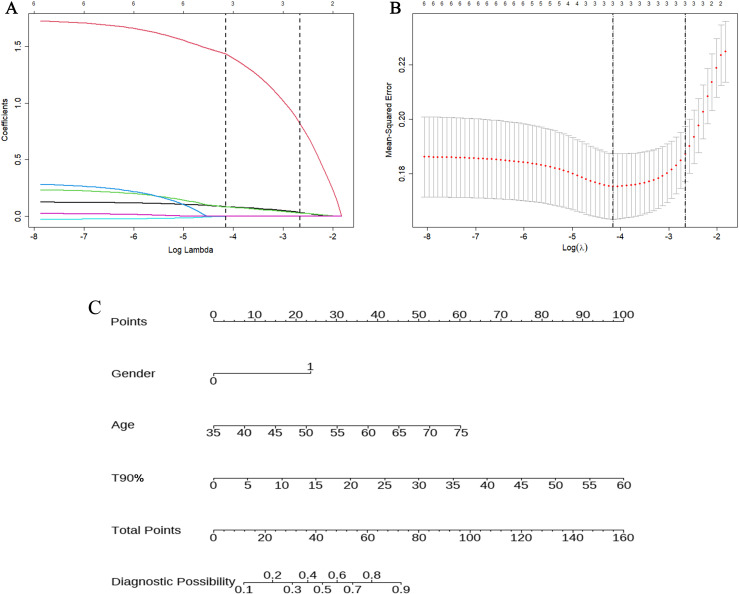
The best matching variables were selected using LASSO regression in the training set. LASSO regression path diagram for variable selection (A). The optimal penalization coefficient lambda was generated in the LASSO regression by the ten-fold cross-validation, the lambda 1se value was selected (B). Nomogram of the prediction model for olfactory disorder in obstructive sleep apnea patients (C).

Univariate analysis in the training set demonstrated significant differences in the six variables between patients with OSA with and without OD, including the variables age, gender, minimum SpO2%, average blood oxygen, ODI, and T90%. The results are presented in Table 2. LASSO regression was used to further analyze the six variables, which identified age, sex, and T90% as the optimal matching factors. The LASSO regression path plot and visualization of variable selection for the best model are depicted in Fig 2A and 2B, respectively.

**Table 2:** Univariate analysis between OSA with OD and OSA without OD in training set.

Variables	Total (n = 87)	Non-OD (n = 29)	OD (n = 58)	*t/z/x²*	*P*
**Age**	53.14 ± 7.24	50.59 ± 5.85	54.41 ± 7.58	-2.6	0.011
**BMI**	27.29 ± 3.69	26.69 ± 3.53	27.58 ± 3.76	-1.09	0.280
**NC**	39.54 ± 4.98	39.57 ± 4.17	39.52 ± 5.38	0.04	0.965
**SPSS**	6.55 ± 1.78	6.76 ± 1.75	6.45 ± 1.81	0.77	0.443
**ESS**	11.31 ± 5.17	11.59 ± 4.86	11.17 ± 5.36	0.36	0.719
**OAI**	7.20(2.30, 18.80)	4.40(0.75, 17.05)	8.70(2.75, 19.63)	1.58	0.114
**CAI**	0.20(0.10, 0.70)	0.30(0.10, 1.35)	0.20(0.10, 0.70)	0.52	0.604
**MAI**	0.10(0.10, 0.80)	0.10(0.10, 0.60)	0.25(0.10, 2.55)	1.48	0.138
**HI**	10.60(6.40, 17.10)	9.10(5.90, 18.15)	11.00(7.00, 17.03)	1.00	0.315
**AHI**	26.00(15.50, 43.60)	20.20(9.75, 35.30)	33.35(16.48, 47.30)	1.79	0.073
**T90%**	4.80(0.30, 10.30)	0.40(0.10, 4.25)	5.95(1.58, 18.35)	3.78	<0.001
**ODI**	25.80(13.60, 45.60)	19.80(9.15, 32.55)	31.80(15.45, 47.28)	2.04	0.042
**Minimum SpO2%**	80.00(75.00, 86.00)	85.00(79.00, 87.00)	79.00(73.00, 84.00)	2.97	0.003
**Average blood oxygen**	94.00(93.00, 95.00)	94.00(94.00, 96.00)	94.00(92.00, 95.00)	2.10	0.036
**ADH**	30.23 ± 7.01	31.00 ± 8.71	29.84 ± 6.04	0.64	0.522
**ADOA**	26.87 ± 9.45	25.21 ± 10.34	27.70 ± 8.94	-1.11	0.274
**Gender**				10.17	0.001
** Female**	21	13(61.9%)	8(38.1%)		
** Male**	66	16(24.2%)	50(75.8%)		
**Hypertension**				2.35	0.125
** Yes**	37	9(24.3%)	28(75.7%)		
** No**	50	20(40.0%)	30(60.0%)		
**Coronary heart disease**				1.45	0.229
** Yes**	15	3(20.0%)	12(80%)		
** No**	72	26(36.1%)	46(63.9%)		
**Hyperlipidemia**				0.01	>0.999
** Yes**	27	9(33.3%)	18(66.7%)		
** No**	60	20(33.3%)	40(66.7%)		
**TDI**	28.25(24.50, 31.50)	33.50(31.50, 35.00)	25.87(22.50, 28.31)	7.57	0.001
** T**	5.94 ± 3.14	8.75 ± 2.97	4.53 ± 2.12	6.82	<0.001
** D**	10.21 ± 2.40	11.90 ± 1.54	9.36 ± 2.31	6.08	<0.001
** I**	12.00(11.00, 13.00)	13.00(12.00, 14.00)	11.00(10.00, 12.00)	4.90	<0.001

Abbreviation: OSA: obstructive sleep apnea; OD: olfactory disorder; BMI: body mass index; NC: neck circumference; SPSS: Subjective perception of sleep score; ESS: Epworth sleep scale; CAI: central apnea index; OAI: obstructive apnea index; MAI: mixed apnea index; HI: hypopnea index; AHI: apnea hypopnea index; T90%: time spent with oxygen saturation below 90%; ODI: 3% oxygen desaturation index; ADH: average duration of Hypopnea; ADOA: average duration of obstructive apnea; TDI: threshold discrimination identification; T: threshold; D: discrimination; I: identification. *P <* 0.05 is statistically significant.

### Multivariate logistic regression analyses in patients with OSA with OD and non-OD

The optimal matching factors were subsequently included in multivariate logistic regression analysis. The results indicated that age (odds ratio [OR], 1.114; 95% confidence interval [CI], 1.029–1.221; *P* =  0.012), sex (OR, 5.434; 95% CI, 1.667–19.98; *P* =  0.007), and T90% (OR, 1.127; 95% CI,1.035–1.276, *P* =  0.024) were determined to be independent factors influencing OD in patients with OSA (Table 3).

**Table 3:** Multivariate logistic regression analysis of features associated with OD in OSA patients of the training set.

Variables	B	SE	OR (95%CI)	*z*	*P*
**Intercept**	-6.826	2.446	0.001 (5.818-0.094)	-2.79	0.005
**Age**	0.108	0.043	1.114 (1.029-1.221)	2.508	0.012
**Gender**	1.693	0.626	5.434 (1.667-19.980)	2.703	0.007
**T90%**	0.120	0.053	1.127 (1.035-1.276)	2.263	0.024

Abbreviation: OD: olfactory disorder; OSA: obstructive sleep apnea; B: regression coefficient; SE: standard error; OR: odds ratio; CI: confidence interval; T90%: time spent with oxygen saturation below 90%. *P <* 0.05 is statistically significant.

### Development of the nomogram prediction model

To predict the risk of OD in patients with OSA, we developed a nomogram model incorporating three independent influencing factors, based on the results of multivariate logistic regression analysis (Fig 2C). A nomogram is a graphical representation in which the variables in a known function relationship are proportionally depicted as scaled straight lines on the same plane. A vertical line was drawn from the corresponding information for each patient to the top points, yielding a score for that patient in a given variable. Then, the sum of the scores for each variable was calculated; the predicted risk corresponding to the summed score was the probability of a diagnosis of OD.

### Validation of discrimination and calibration of the nomogram model

**Fig 3:**
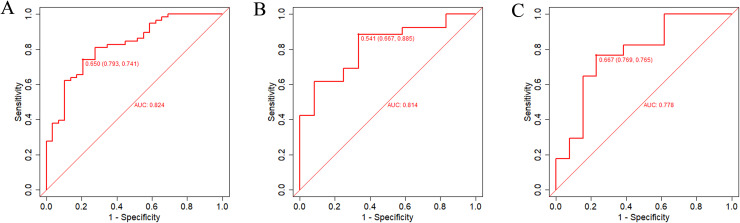
The receiver operating characteristic (ROC) curves of the model in the training set (A), internal validation set (B), and external validation set (C).

The discrimination of the nomogram was validated using ROC curves. The results exhibited good discriminative performance in distinguishing patients with OSA who had OD from those who did not have OD, with an AUC of 0.824 (95% CI, 0.733–0.915; Fig 3A), 0.814 (95% CI: 0.673–0.955; Fig 3B), and 0.778 (95% CI: 0.601–0.955; Fig 3C) in the training set, internal validation set, and external validation set, respectively.

**Fig 4:**
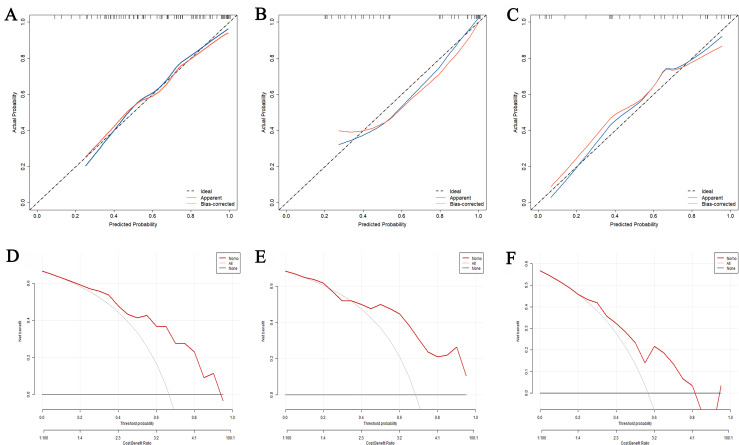
The calibration curves of the model in the training set (A), internal validation set (B), and external validation set (C). The decision curve analysis of the model in the training set (D), internal validation set (E), and external validation set (F).

In the calibration curve, the predicted probabilities of the model were plotted on the X-axis, and the actual probabilities observed were plotted on the Y-axis. The 45° black dashed line represented the ideal prediction, the red solid line represented bias-corrected prediction with bootstrapping 500 repetitions, and the blue solid line represented the training set (Fig 4A), internal validation set (Fig 4B), and external validation set (Fig 4C). A closer alignment between the blue solid line and black diagonal dotted lines indicates better prediction performance. The figure shows that the blue predicted line overlapped well with the black ideal reference line in the three groups.

The Hosmer–Lemeshow test revealed that there was no significant difference between the predicted and observed probabilities of OD in the training group (R2 =  0.382, *P* =  0.201; Fig 4A), internal validation set (R2 =  0.379, *P* =  0.305; Fig 4B), and external validation set (R2 =  0.211, *P* =  0.640; Fig 4C), suggesting good calibration performance of this nomogram model.

### Decision curve analysis (DCA) of the nomogram prediction model

DCA was used to evaluate the net benefits of the diagnostic nomogram model to further validate its clinical utility. The results revealed that the nomogram model was applicable and gained the maximum net benefits when the threshold probability in the training set, internal validation set, and external validation set was 0.15–0.94 (Fig 4D), 0.11–0.99 (Fig 4E), and 0.21–0.81 (Fig 4F), respectively.

## Discussion

The incidence of OD in patients with OSA is 72.4–84% [7,15]. OD not only has a variety of detrimental effects on individuals but is also linked with adverse prognostic implications. Therefore, olfactory assessment is important as part of routine examination in patients with OSA. However, olfactory assessment is constrained by its inherently time-consuming and labor-intensive limitations. Herein, we developed and validated a nomogram model based on sex, age, and T90% in an internal and external cohort. Our nomogram offers a quick and reliable method to screen for olfactory dysfunction in patients with OSA, aiding in early detection and targeted care.

The strengths and innovations of this study are reflected in several key aspects. First, we developed a nomogram using data from two different hospitals, ensuring a broad and representative sample, which allows for more precise estimates and narrower CIs. Moreover, our nomogram provides a graphical representation of a statistical diagnostic model, predicting the likelihood of olfactory dysfunction in patients with OSA based on easily accessible clinical variables. In addition, using continuous variables instead of categorical ones, we enhanced the model’s ability to deliver personalized and stratified predictions, improving its clinical applicability.

In our study, we identified male sex and older age as high-risk factors for OD among patients with OSA. This finding is consistent with those of other studies suggesting that women generally outperform men in tests of odor detection, identification, and discrimination [24–26]. Similarly, most functional imaging and electrophysiological studies also suggest that, when present, sex differences tend to favor women [27]. The underlying biological basis for these sex differences may be linked to variations in the circulating level of reproductive hormones between men and women [27]. However, it is important to note that the gap in olfactory capabilities between the sexes tends to narrow with advancing age [28]. This could be attributed to age-related alterations within the entire olfactory circuitry, as well as a decrease in neurogenesis related to olfaction as age increases [29]. In addition, a meta-analysis suggested that sex differences in odor identification were primarily observed in younger adults (18–50 years old), but these differences became negligible in older individuals over the age of 50 years [30]. Despite progress in this area, it is still difficult to fully understand the reasons for the decline in olfactory function owing to age and sex differences. Our findings are consistent with those of other studies that have identified a link between OD and OSA-associated chronic intermittent hypoxia (CIH), a predominant feature of OSA [31]. Significant correlations have been observed between OD and CIH in patients with OSA [15]. Moreover, studies involving mice with CIH have shown changes in the neural networks of the main olfactory bulb, which might account for reports of OD in patients with OSA [32]. CIH-induced alterations in interneurons may extend to the granule cell network within the olfactory bulb, leading to diminished functional activity [33].

OD is an independent risk factor that can negatively impact cognitive functions. There is a 15-fold increase in the risk of developing cognitive impairment among people with OD, compared with those who have no baseline sensory impairments [34]. Notably, OD may not be permanent. Olfactory perception is eminently plastic and can be reversed by targeted olfactory training. The olfactory system is highly responsive to training, which may facilitate the transfer of learning to other sensory domains [35].

Our nomogram can serve as an effective tool in screening for OD among patients with OSA. The nomogram offers a simpler and more sophisticated tool with multiple advantages. By integrating patient- and disease-specific characteristics, nomograms can provide personalized risk assessment, which offers a notable advantage [36]. In addition, nomograms can incorporate continuous variables and important disease determinants into the diagnostic processes. In this study, we provide a user-friendly tool for clinical decision-making in the management of OSA.

Certain issues must be emphasized in the present study. First, although including samples from different populations at two study hospitals may reflect the actual rate of OD among patients with OSA, the overall sample size in this study was relatively small. Second, this was a retrospective study, and we did not have access to data on all PSG parameters in the cohort, especially the sleep segments. Other scholars have considered that sleep fragmentation is an important mechanism contributing to the pathophysiology of OD in individuals with OSA [7]; however, that past study was also limited by a small sample size. In other words, our nomogram uses data that are readily accessible in clinical settings and even obtainable through portable devices, thereby circumventing limitations in clinical application. Consequently, further studies with larger samples and more comprehensive data are necessary to validate and expand our findings.

In conclusion, considering the substantial number of patients with OSA who present with OD, the present developed nomogram offers a straightforward and user-friendly assessment tool. Our nomogram exhibited improved predictive accuracy and demonstrated good performance in the external validation cohort. This tool can enable more accurate stratification and improved identification of OD, consequently facilitating risk-based management of patients with OSA in clinical practice.

## Supporting information

S1 File Raw data for the study. This file contains the raw data collected during the study, including all measurements and observations. The data are organized in separate sheets for each experiment, with detailed column headers explaining the variables.
(XLSX)
